# Deep Learning Empowers Endoscopic Detection and Polyps Classification: A Multiple-Hospital Study

**DOI:** 10.3390/diagnostics13081473

**Published:** 2023-04-19

**Authors:** Ming-Hung Shen, Chi-Cheng Huang, Yu-Tsung Chen, Yi-Jian Tsai, Fou-Ming Liou, Shih-Chang Chang, Nam Nhut Phan

**Affiliations:** 1 Department of Surgery, Fu Jen Catholic University Hospital, Fu Jen Catholic University, New Taipei City 24205, Taiwan; 2 School of Medicine, College of Medicine, Fu Jen Catholic University, New Taipei City 24205, Taiwan; 3 Department of Surgery, Taipei Veterans General Hospital, Taipei City 11217, Taiwan; 4 Institute of Epidemiology and Preventive Medicine, College of Public Health, National Taiwan University, Taipei City 10663, Taiwan; 5 Department of Internal Medicine, Fu Jen Catholic University Hospital, New Taipei City 24205, Taiwan; 6 Division of Colorectal Surgery, Department of Surgery, Fu Jen Catholic University Hospital, New Taipei City 24205, Taiwan; 7 Graduate Institute of Biomedical Electronics and Bioinformatics, Department of Electrical Engineering, National Taiwan University, Taipei City 10663, Taiwan; 8 ASUSTeK Computer Inc., Taipei City 11259, Taiwan; 9 Division of Colorectal Surgery, Department of Surgery, Cathay General Hospital, Taipei City 106443, Taiwan; 10 Bioinformatics and Biostatistics Core, Centre of Genomic and Precision Medicine, National Taiwan University, Taipei City 10055, Taiwan

**Keywords:** colorectal cancer, endoscopic, deep learning

## Abstract

The present study aimed to develop an AI-based system for the detection and classification of polyps using colonoscopy images. A total of about 256,220 colonoscopy images from 5000 colorectal cancer patients were collected and processed. We used the CNN model for polyp detection and the EfficientNet-b0 model for polyp classification. Data were partitioned into training, validation and testing sets, with a 70%, 15% and 15% ratio, respectively. After the model was trained/validated/tested, to evaluate its performance rigorously, we conducted a further external validation using both prospective (*n* = 150) and retrospective (*n* = 385) approaches for data collection from 3 hospitals. The deep learning model performance with the testing set reached a state-of-the-art sensitivity and specificity of 0.9709 (95% CI: 0.9646–0.9757) and 0.9701 (95% CI: 0.9663–0.9749), respectively, for polyp detection. The polyp classification model attained an AUC of 0.9989 (95% CI: 0.9954–1.00). The external validation from 3 hospital results achieved 0.9516 (95% CI: 0.9295–0.9670) with the lesion-based sensitivity and a frame-based specificity of 0.9720 (95% CI: 0.9713–0.9726) for polyp detection. The model achieved an AUC of 0.9521 (95% CI: 0.9308–0.9734) for polyp classification. The high-performance, deep-learning-based system could be used in clinical practice to facilitate rapid, efficient and reliable decisions by physicians and endoscopists.

## 1. Introduction

Colorectal polyps refer to tissue on the surface of the large intestinal mucosa that is formed by abnormal hyperplasia of the epithelium [1]. Polyps are divided into two main categories, namely, hyperplastic and adenomatous polyps. Clinically, 95% of polyps are of these types, with distinct properties and treatment modalities [2]. Hyperplastic polyps most frequently appear in the sigmoid colon and rectum and display tiny mucosal protrusions with a flat shape. Their color is usually the same as the surrounding mucosa or slightly whiter. The probability of these polyps transforming into a malignant tumor in the future is very low. Adenomatous polyps need to be inspected carefully due to their role as precursors of cancer, and they can grow in various parts of the large intestine [3]. Adenomatous polyps can be further divided into tubular, villous and mixed types upon pathological inspection of the tissue. Among these polyps, villous adenomas are more likely to become cancerous [4].

In Taiwan, more than 15,410 new cases of colorectal cancer (CRC) were diagnosed in 2016. The annual incidence rate of CRC is 0.044%, and the mortality rate is 0.015% in both sexes [5]. CRC and adenomatous polyps have an incidence rate of 3–5% in subjects with a positive fecal occult blood test. This group has a higher risk of CRC, and colonoscopy is a useful method for cancer detection. In addition to early detection of cancer by colonoscopy (as mentioned, there is 1 CRC detected in every 20 to 30 invitees, and half are carcinoma in situ or first-stage cancer), CRC can be prevented by removing adenomatous polyps found during examination. Therefore, colonoscopy can not only reduce mortality, but can reduce the incidence of CRC. According to research in the United States, colonoscopy can reduce the mortality rate of CRC by 65–75% [6].

Colonoscopy is considered the gold standard for polyp detection and has the additional benefit of allowing the removal of any suspicious polyps [7]. CRC is preventable by removing polyps in the large intestine, and it is estimated that with a 1% increase in polyp detection, the incidence of CRC could be further reduced by 3% [8]. Unfortunately, previous studies have revealed that an average of 22% to 28% of polyps and 20% to 24% of potentially cancerous adenomas can be missed by colonoscopy examination [9]. Polyps can be missed due to their uncommon (flat and small) shape and/or operator error. Therefore, there remains a need to develop a computer-aided system to assist endoscopists to improve the polyp detection rate and adenoma classifications. At present, an improvement in the imaging system for colonoscopy can be made by a switch to the use of Narrow Band Imaging (NBI) [10]. NBI improves the observation of capillaries and fine structures on the mucosal surface when operating the endoscope. It also assists in identifying whether the polyp is cancerous. Using NBI, polyps can be classified according to the Japan NBI Expert Team (JNET) classification [11], predicting the cancerous progression of polyps and assisting physicians in deciding whether endoscopic resection or surgery is required.

In the last five years, machine learning (ML) models have been extensively applied to biomedical research [12,13]. A subdomain of ML, known as deep learning (DL), has demonstrated its powerful prediction capacity, as long as the required amount of data is fulfilled, which varies extensively according to the targeted application [14]. DL models contain the data transformation processes within their architecture. The features/variables are automatically extracted and used for model training. There are several common DL architectures, which have demonstrated a high capacity for biomedical tasks, such as Convolutional Neural Network (CNN) [14,15,16], Recurrent Neural Network (RNN) [17] and Deep Belief Network (DBN) [18]. Furthermore, studies using DL methods applied to colonoscopy images have attracted considerable attention recently [19]. In 2019, Yamada et al. [20] developed a real-time endoscopic image diagnosis tool for colonoscopy images with high accuracy using a small dataset. In 2020, working on a larger-scale dataset of 27,598 images from both normal and polyps images, Ozawa et al. [21] applied DL techniques with the CNN model for the detection and classification of polyps. Colorectal polyp detection against normal mucosa has also been completed using ZF-Net [22]. Furthermore, apart from detection and classification tasks using colonoscopy images, a segmentation approach has also been done with u-net and a dilation convolution method [23]. However, a DL model’s performance is not only affected by the model architecture, but it also dramatically relies on the data quality [24] and the amount of data [25]. To fill the gap of the huge amount of data needed to train and test the DL model, in the current study, we aimed to leverage the huge dataset from multiple hospitals to enhance the DL model’s accuracy and expose the models to diverse data. In addition, we benchmarked the model classification results against those of a team of pathologists, who used the patient pathology reports as the golden standard for assessing adenomatous versus non-adenomatous polyps.

The DL model was developed to enhance diagnostic accuracy and reduce the workload of physicians, making endoscopy examinations more efficient and safer. Colonoscopy based on DL can help to improve ADR (i.e., adenoma detection rate, glandular polyp detection rate) by extracting the features of the polyps to assist physicians in determining the most optimal treatment of polyps. We named the DL model developed EndoAIM^TM^. EndoAim^TM^ can assist trained gastroenterology and surgery clinicians in the process of endoscopy, complete the inspection of polyps, reduce the missed polyp rate during the examination and classify their characteristics.

## 2. Materials and Methods

### 2.1. Data Collection Procedures

Colonoscopy images were collected from 5000 patients, including images of the normal background, general bowel and polyps, at Fu-Jen Catholic University Hospital (FJUH), using the Picture Archiving and Communication System (PACS), between 1 September 2017 and 30 September 2020. The colonoscopy images were manually reviewed to remove any low-quality images. Blurry images were verified for contrast, underexposure or overexposure. Low-contrast images were also removed.

In total, 430,921 images were collected for the detection task, and about 256,220 images remained after the preprocessing steps. We used 85% of the data (218,720 images) to do the fivefold cross-validation and the remaining 15% for the test set. For the classification task, we collected a total of 17,485 images, and 5394 images remained after the preprocessing steps.

**Figure 1:**
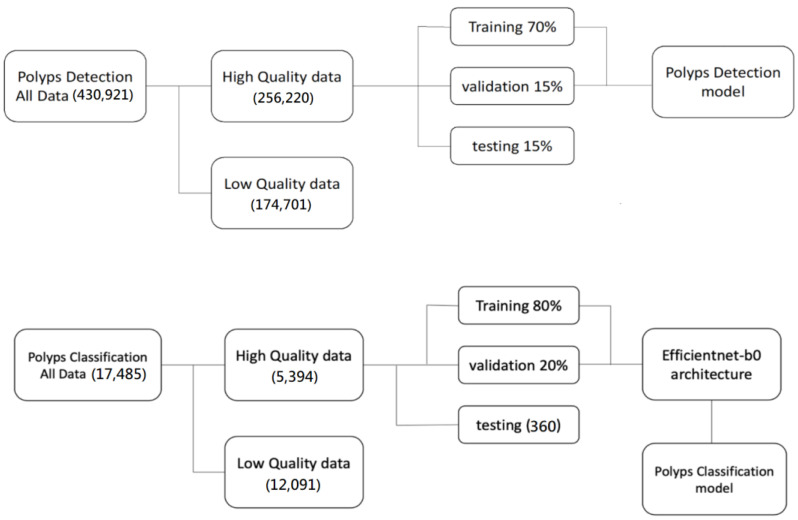
Flowchart illustrating the polyp detection and classification approach for the proposed model.

We used these 5394 images to do a fivefold cross-validation. With the help of the IT department, all patients’ information, such as name, gender, age, address, date of birth, ID number, medical record number, health insurance card number, phone number and e-mail address, was delinked and masked to maintain patient privacy. The overall workflow of the model training is depicted in Figure 1.

### 2.2. Polyp Annotation and Classification Protocol

Collected images containing polyps were submitted to three colorectal surgeons/gastroenterologists (M.H.S., C.K.C. and Y.T.C.) for polyp annotation and classification according to NBI images, with the intention that all three physicians would agree on the annotated areas and polyp classification. Images were only included in the DL model analysis if at least two physicians had similar interpretations for a polyp.

### 2.3. Deep Learning Algorithms

The collected de-identified images, including images of the general background, normal intestine and annotated polyps, were used to develop the DL-based system.

#### 2.3.1. Model Architecture

##### Polyps Detection Model

The detection model was developed based on CNN. We selected stochastic gradient descent as the model optimizer, with the initial learning rate at 0.01 and weight decay at 5 × 10^−4^. The box loss gain and class loss gain were set at 0.05 and 0.5, respectively.

##### Polyp Classification Model

We used the EfficientNet-b0 model architecture [26] with weight from ImageNet for the transfer learning protocol. The model optimizer was Adam, with an initial learning rate at 1 × 10^−4^ and weight decay at 0. The model was trained for 150 epochs.

For the detection model selection, we conducted a pilot study using 10,000 colonoscopy polyp images downloaded from https://datasets.simula.no/kvasir/ (accessed on 1 March 2020). The results confirmed CNN was the best performer regarding the speed and accuracy of object detection.

##### Model Validation

After completing the deep learning, the polyp and NBI images that were collected and de-identified, yet not used in the deep learning, were used for validation. (About 1250 images were examined; these images had been previously marked and analyzed by three physicians). The verification method was to let the DL model classify the 1250 images, and the target accuracy rate was >95%.

##### Evaluation Metrics

We used polyp detection accuracy, mean average precision (mAP), sensitivity, specificity and the receiver operating characteristic curve (ROC) to evaluate the performance of the DL-based system in both the validation and testing sets.

### 2.4. Graphic User Interface for Model Deployment

The model was deployed into a graphical user interface (GUI) as a final product. Representative images were selected from the illustration with the GUI. Panel A displaying the polyp detection system had detected a polyp and placed a green square around the area. When a polyp appeared on the endoscopic screen, it would be immediately marked to remind the physicians regarding the location of the polyp. The EndoAim^TM^ sends alerts and notes to the endoscopist. A yellow warning map was shown when a polyp was identified on the screen, and it appeared for two seconds. The purpose was to reinforce the intensity of the reminder to the operator that polyps were detected within the range in order to avoid their neglect. In clinical practice, a DL model might detect polyps and mark them with a screen flash, and the physician might fail to immediately recognize the targeted lesions and miss them. To avoid this shortcoming, a new thumbnail reminder function was added to remind the operator of the latest polyp detected. These images were for the endoscopists’ reference. Polyp snapshots were only displayed on the screen and were not stored permanently. When the DL model detected any polyps, the thumbnail was displayed. Next, if there were no polyps detected on the screen, a countdown of two seconds began. Thereafter, there were two situations: (1) If in two seconds, other polyps were detected, then nothing happened, and the thumbnail did not change; (2) After two seconds, if other polyps were detected, then the thumbnail updated.

Currently, when a physician performs a colonoscopy and wants to classify polyps, he must bring the endoscopic lens closer to the polyp and turn on the “NBI” function so that the targeted lesion can be clearly identified.

### 2.5. Hardware

All the data processing and model training were conducted in a system with a CPU i7-10700k, GPU 3080ti and 128 GB RAM, using the Pytorch platform.

### 2.6. External Validation Using Multiple-Hospital Datasets from Both Prospective and Retrospective Approaches

After the model had been trained and tested with the dataset from FJUH, we performed external validation using a multiple-hospital dataset collected from prospective and retrospective data collections.

In the prospective mode, we collected 150 colonoscopy videos from 150 patients from 3 hospitals, resulting in 516 polyps. This dataset was used for the polyp detection mode. Each hospital included 50 colonoscopy videos. A routine colonoscopy was performed by a specialist physician. During the inspection, the results of the EndoAim^TM^ detection of polyps and the doctor’s judgment were recorded, and the frame Ground Truth was marked after the inspection; the EndoAim^TM^ polyp detection results were compared with the ground truth to calculate the EndoAim^TM^ polyp detection sensitivity (lesion-based sensitivity) and the Frame-based specificity results.

In the retrospective mode, we collected 385 NBI images and single polyp photos taken by various hospitals using Olympus CV290 series products, together with a matching pathology report. There were 193 and 192 images of the non-adenoma and the adenoma groups, respectively. The pathological report result from each patient was considered as the ground truth, then input into the EndoAim^TM^ model for prediction. AUC and the sensitivity and specificity of the EndoAim^TM^ adenoma classification for each polyp was used to evaluate the model performance.

## 3. Results

### 3.1. Deep Learning Detection Model Performance

The model performance for polyp detection was evaluated using standard metrics, such as sensitivity, specificity, AUC score and mAP. The model achieved 0.9709 (95% CI: 0.9646–0.9757) sensitivity and 0.9701 (95% CI: 0.9663–0.9749) specificity (Table 1). The AUC score of the model reached the state-of-the-art level of 0.9902. When evaluated for the intersection over the union for lesion detection, the model approached 0.8845 for the mAP metric (Table 1). All of these metrics were generated with the testing set.

### 3.2. High Sensitivity and Specificity Deep Learning Classification Model

After the polyps were detected using the DL model, we used the classification model to distinguish adenomatous versus non-adenomatous polyps. The classification model performance also provided extremely high sensitivity (0.9889), specificity (0.9778) and F1 score (0.9834). The AUC score of the model reached a state-of-the-art level of 0.9989 (CI: 0.9954–1.00) (Table 2).

### 3.3. Illustration of the Deployed Deep Learning Models into GUI

**Figure 2:**
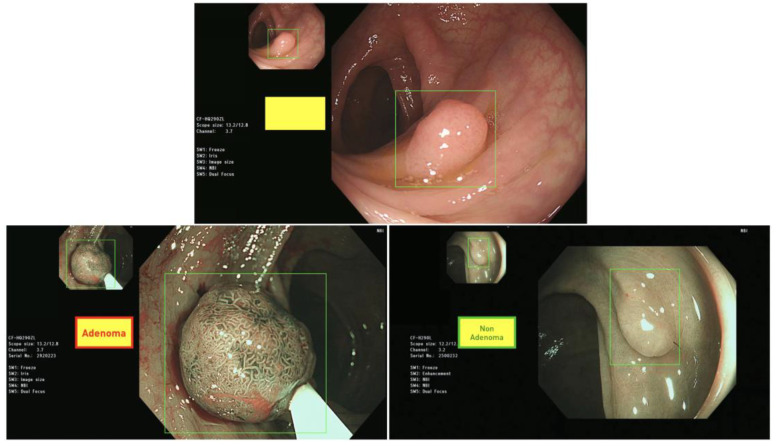
Demonstration of the EndoAIM^TM^ system using testing dataset.

We deployed the detection and classification models into the GUI and named the system EndoAim^TM^. The results of the real-time detection and classification are illustrated in Figure 2.

Polyps are classified as follows: (a) the length and width of the polyp marking box are greater than 20% of the length and width of the display range of the colonoscopy and maintained for 2 seconds; (b) the screen switches to the NBI display; (c) the picture must be in focus. The classification results reveal non-adenomatous polyps/adenomatous polyps (Figure 2).

### 3.4. Model Benchmark Using Multiple Hospital Datasets

The model performance using external datasets was conducted in prospective and retrospective modes using datasets obtained from three hospitals.

#### 3.4.1. Prospective Datasets for Polyp Detection Testing

For the former approach, the model performance for polyp detection reached an average of 0.9516 (CI: 0.9295–0.9670) using a lesion-based sensitivity of 0.9817, 0.9389 and 0.9360 for hospitals A, B and C, respectively. Additionally, the model performance with frame-based specificity was at 0.9720 (CI: 0.9713–0.9726) for the average of the three hospitals, with 0.9676, 0.9833 and 0.9605 for hospitals A, B and C, respectively (Table 3).

#### 3.4.2. Retrospective Datasets for Polyp Classification Testing

The model performance for polyp classification achieved an average AUC score of 0.9521 (CI: 0.9308–0.9734). The highest AUC (0.9947 (CI: 0.9817–1.00)) was obtained for the hospital A data, whereas the lowest AUC (0.9207 (CI: 0.8749–0.9665)) was obtained for the hospital C data (Table 4).

## 4. Discussion

In the current study, we developed two high-performance DL models for polyp detection and classification of detected polyps into adenomatous and non-adenomatous types in CRC patients. We further deployed the models into a GUI that directly connected to the endoscopic system. The system assisted physicians in their routine work, with high accuracy in both detecting and classifying adenomatous and non-adenomatous polyps. To our knowledge, this is the first time a system has been developed using multiple datasets, including prospective and retrospective data collections, with the capability to detect polyps (lesion-based sensitivity at 0.9516, frame-based specificity at 0.9720) and distinguish adenomatous from non-adenomatous polyps (AUC at 0.9521) using the pathology reports. The developed model should improve the colonoscopy procedure for polyp detection and classification and facilitate higher precision and efficiency for physicians.

The development of AI technology, particularly DL algorithms, has contributed to the advancement of biomedical research and wider applications in recent decades [12,13,27]. Computer-aided detection (CAD) systems based on DL models trained on imaging datasets are attracting the particular attention of biomedical researchers. Most CAD systems are not developed as stand-alone tools but are usually integrated or deployed into devices for clinical applications. Polyp detection and classification is a field in which CAD systems have been extensively studied by both ML and conventional approaches, and various models/tools have been proposed in this competitive research domain [22,27,28,29,30]. The critical metrics to evaluate a model’s performance for polyp detection and classification are usually based on the sensitivity and specificity for polyp localization and the classification rate for adenomatous versus non-adenomatous polyps. In the current study, our DL models achieved a state-of-the-art sensitivity and specificity of 0.9709 and 0.9701, respectively, for the detection model. The adenoma classification also reached a high AUC value of 0.9989. The missing rate of adenoma classification, especially the interobserver variability, is noteworthy [31]. In our study, we recruited three experienced endoscopists to evaluate the images, and we sought to obtain consensus on the polyp areas that were eventually used for the DL model training and evaluation. Training the models using rigorously selected images should enable them to prevent or reduce the false-positive or false-negative rates when used by physicians for real-time colonoscopy monitoring. The system should also help reduce the detection and procedure times for physicians.

Furthermore, although the screening guidelines for CRC indicate the necessity for removing precancerous polyps, this process is highly dependent on the experience of the colonoscopy practitioner [32]. Therefore, a DL-based system could play a crucial role in improving the screening policy and could leverage less experienced physicians for the efficient detection and classification of polyps. Although the final decision should be made by the operator, with the assistance of the DL systems, it could be made with increased confidence.

An increasing number of applications using DL algorithms, particularly CNN, have been described recently. Leveraging the pretrained models on the ImageNet dataset [33], this pretrained model has been shown to boost the model performance significantly and reduce training times [34,35,36]. CNN models have also been used for polyp detection and classification and reached a high level of accuracy for both static images [37,38] and video data [39]. Nevertheless, although a plethora of models has been developed and high sensitivity/specificity metrics obtained, the amount of data used to train these models has usually been limited to both publicly available datasets—such as CVC-CLINIC [40], ETIS-LARIB [41], CVC-ClinicVideoDB [41], CVC-ColonDB [42], CVC-EndoSceneStill [43], CVC-Clinic2015 and CVC-Clinic2015—and in-house datasets from different health institutions [40,44,45]. Although our study also collected a dataset from a single hospital, the amount of data was large (256,220 images from 5000 patients) compared with that in these other datasets. In addition, 10,000 colonoscopy polyp images from the Kvasir dataset were used to conduct a pilot training before using our in-house hospital data, which could partially serve as another independent dataset for the model development [46].

Together with the evolving DL algorithms, the number of endoscopic datasets, including static image and video data, is increasing, as is their quality and size. In addition, various publicly available datasets can be easily accessed and downloaded and are usually relatively small in size; but unfortunately, the in-house datasets from different health institutions have not been opened up to public access, which hinders efforts to benchmark DL and ML models properly. Although researchers can compare the model performance using common metrics, such as accuracy, sensitivity and specificity, as well as F1 score and AUC-ROC score, the lack of common datasets that have been used to develop and test the models has rendered head-to-head comparisons difficult. For instance, a model trained and tested on a larger dataset with marginally lower accuracy than a model trained and tested with a smaller dataset could not be translated directly into the model performance benchmarks using the common metrics mentioned.

Our study used a large dataset of patient numbers and total number of images and reached high sensitivity and specificity. In a previous study, an AI system developed for detecting polyps reached 97.3% sensitivity and 99.0% specificity [20], which is slightly better than our model performance. However, the dataset (705 images from 752 lesions) was smaller in size than ours (2000 images). In another study using a large dataset with 27,598 images, including colorectal polyps and normal images, the CNN model reached 90% sensitivity and an 83% positive predictive value for the white light images and 97% and 98% with NBI images [21]. Another study by Sun et al. [23] developed a model using public datasets and obtained F1 scores of 96.11% and 80.86% with the CVC-ClinicDB (612 images from colonoscopy sequences) and ETIS-Larib Polyp DB (196 images) datasets, respectively. Yet another study obtained extremely high accuracy (98%) using 11,300 images of colorectal polyps and normal mucosa [22] by employing a modified version of ZF-Net [47]. In addition to the use of DL models and endoscope images, polyp detection systems have been developed using ML models, such as random forest and CT images. This model was developed using a small dataset of 169 images from 107 colorectal polyps from 63 elderly patients. This model displayed a sensitivity of 82% and a specificity of 85% using a hold-out test set with only 118 images [48].

In summary, with the evolution of DL and ML models in colorectal polyp detection and classification, excellent performance has emerged from both public and private datasets. These achievements should transform the healthcare management and screening policy in this domain soon. With numerous models being developed, there is a need for the development of specific regulations and policies for using these DL-based systems in the clinical setting.

## 5. Conclusions

In our study, we have developed DL-based systems for the detection and classification of polyps using a large hospital-based dataset. The models attained a state-of-the-art sensitivity and specificity in validation and testing sets, and they have been deployed into an Olympus CV290 machine, which is ready to use in clinical practice.

Although we have developed a DL model using a relatively large dataset and tested it rigorously with an external validation dataset from both prospective and retrospective approaches, the external validation sets were relatively small compared with the training dataset. The small validation size does not sufficiently reflect the model’s robustness and accuracy. Therefore, in a prospective study, our team will recruit more patients from different health institutions for external evaluation to reduce this limitation.

## Figures and Tables

**Table 1:** Evaluation of deep learning detection model.

Sensitivity (95% CI)	0.9709 (0.9646–0.9757)
Specificity (95% CI)	0.9701 (0.9663–0.9749)
AUC	0.9902
mAP 0.5:0.95	0.8845

**Table 2:** Evaluation of deep learning classification model.

Sensitivity	0.9889
Specificity	0.9778
AUC (95% CI)	0.9989 (0.9954–1.00)
F1 score	0.9834

**Table 3:** Model benchmark using prospective approach for polyps detection.

Parameters	Hospital A	Hospital B	Hospital C	3 Hospitals
Lesion-based Sensitivity (95% CI)	0.9817 (0.9476, 0.9938)	0.9389 (0.8939, 0.9655)	0.9360 (0.8891, 0.9639)	0.9516 (0.9295–0.9670)
Frame-based Specificity (95% CI)	0.9676 (0.9665, 0.9687)	0.9833 (0.9824, 0.9840)	0.9605 (0.9589, 0.9620)	0.9720 (0.9713–0.9726)

**Table 4:** Model benchmark using retrospective approach for classification.

Parameters	Hospital A	Hospital B	Hospital C	3 Hospitals
AUC (95% CI)	0.9947 (0.9817–1.00)	0.9497 (0.9123, 0.9871)	0.9207 (0.8749, 0.9665)	0.9521 (0.9308, 0.9734)

## Data Availability

Data for the pilot study using 10,000 colonoscopy polyp images downloaded from https://datasets.simula.no/kvasir/ (accessed on 1 March 2020).
